# Contact Tracing Assessment of COVID-19 Transmission Dynamics in Taiwan and Risk at Different Exposure Periods Before and After Symptom Onset

**DOI:** 10.1001/jamainternmed.2020.2020

**Published:** 2020-05-01

**Authors:** Hao-Yuan Cheng, Shu-Wan Jian, Ding-Ping Liu, Ta-Chou Ng, Wan-Ting Huang, Hsien-Ho Lin

**Affiliations:** 1 Epidemic Intelligence Center, Taiwan Centers for Disease Control, Taipei, Taiwan; 2 Institute of Epidemiology and Preventive Medicine, National Taiwan University College of Public Health, Taipei, Taiwan; 3 Office of Preventive Medicine, Taiwan Centers for Disease Control, Taipei, Taiwan; 4 Global Health Program, National Taiwan University College of Public Health, Taipei, Taiwan

## Abstract

**Importance:**

The dynamics of coronavirus disease 2019 (COVID-19) transmissibility are yet to be fully understood. Better understanding of the transmission dynamics is important for the development and evaluation of effective control policies.

**Objective:**

To delineate the transmission dynamics of COVID-19 and evaluate the transmission risk at different exposure window periods before and after symptom onset.

**Design, Setting, and Participants:**

This prospective case-ascertained study in Taiwan included laboratory-confirmed cases of COVID-19 and their contacts. The study period was from January 15 to March 18, 2020. All close contacts were quarantined at home for 14 days after their last exposure to the index case. During the quarantine period, any relevant symptoms (fever, cough, or other respiratory symptoms) of contacts triggered a COVID-19 test. The final follow-up date was April 2, 2020.

**Main Outcomes and Measures:**

Secondary clinical attack rate (considering symptomatic cases only) for different exposure time windows of the index cases and for different exposure settings (such as household, family, and health care).

**Results:**

We enrolled 100 confirmed patients, with a median age of 44 years (range, 11-88 years), including 44 men and 56 women. Among their 2761 close contacts, there were 22 paired index-secondary cases. The overall secondary clinical attack rate was 0.7% (95% CI, 0.4%-1.0%). The attack rate was higher among the 1818 contacts whose exposure to index cases started within 5 days of symptom onset (1.0% [95% CI, 0.6%-1.6%]) compared with those who were exposed later (0 cases from 852 contacts; 95% CI, 0%-0.4%). The 299 contacts with exclusive presymptomatic exposures were also at risk (attack rate, 0.7% [95% CI, 0.2%-2.4%]). The attack rate was higher among household (4.6% [95% CI, 2.3%-9.3%]) and nonhousehold (5.3% [95% CI, 2.1%-12.8%]) family contacts than that in health care or other settings. The attack rates were higher among those aged 40 to 59 years (1.1% [95% CI, 0.6%-2.1%]) and those aged 60 years and older (0.9% [95% CI, 0.3%-2.6%]).

**Conclusions and Relevance:**

In this study, high transmissibility of COVID-19 before and immediately after symptom onset suggests that finding and isolating symptomatic patients alone may not suffice to contain the epidemic, and more generalized measures may be required, such as social distancing.

## Key Points

**Question:**

What is the transmissibility of coronavirus disease 2019 (COVID-19) to close contacts?

**Findings:**

In this case-ascertained study of 100 cases of confirmed COVID-19 and 2761 close contacts, the overall secondary clinical attack rate was 0.7%. The attack rate was higher among contacts whose exposure to the index case started within 5 days of symptom onset than those who were exposed later.

**Meaning:**

High transmissibility of COVID-19 before and immediately after symptom onset suggests that finding and isolating symptomatic patients alone may not suffice to interrupt transmission, and that more generalized measures might be required, such as social distancing.

## Teaser

This study delineates the transmission dynamics of coronavirus disease 2019 (COVID-19) and evaluates the transmission risk at different exposure window periods before and after symptom onset.

## Introduction

The coronavirus disease 2019 (COVID-19) outbreak that originated in Wuhan, China, spread to more than 100 countries within 2 months of when the severe acute respiratory syndrome coronavirus 2 (SARS-CoV-2) was identified in January 2020.^1,2^ Following the Wuhan lockdown and other extreme social-distancing measures conducted by the Chinese government, several countries with widespread outbreaks implemented similar measures, including shutting down entire cities or communities, banning international or domestic travel, conducting border control with symptom screening, and implementing isolation and quarantine.

The unknown epidemiologic characteristics and transmission dynamics of a novel pathogen, such as SARS-CoV-2, complicate the development and evaluation of effective control policies.^3^ The serial interval of COVID-19, defined as the interval between the symptom onset of the index case and that of the secondary case, was found to be short (4-5 days) and was similar to the estimated incubation period.^4^ The short serial interval of COVID-19 and results from viral shedding studies suggested that most transmission occurred near or even before the time of symptom onset.^4,5,6^ On the other hand, prolonged viral shedding raised concerns about prolonged infectiousness of patients and the need for extended isolation. A few preliminary contact-tracing studies showed that the highest-risk exposure setting of COVID-19 transmission was the household.^7,8,9^ Nevertheless, it is not known when and how long a patient with COVID-19 should be isolated or whether close contacts should be quarantined. Additional information is needed about the transmission risk at different time points before and after symptom onset and with different types of exposures, such as through the household or a health care facility.

In Taiwan, the first COVID-19 case was confirmed on January 21, 2020.^10^ With proactive containment efforts and comprehensive contact tracing, the number of COVID-19 cases remained low, as compared with other countries that had widespread outbreaks.^11,12^ Using the contact tracing data in Taiwan, we aimed to delineate the transmission dynamics of COVID-19, evaluate the infection risk at different exposure windows, and estimate the infectious period.

## Methods

### Study Population

On January 15, 2020, in response to the outbreak in Wuhan, the Taiwan Centers for Disease Control (Taiwan CDC) made COVID-19 a notifiable disease. We conducted a prospective case-ascertained study that enrolled all the initial 100 confirmed cases in Taiwan between January 15 and March 18, 2020, and their close contacts. All contacts were followed up until 14 days after the last exposure to the index case. The last follow-up date was April 2, 2020.

The study followed the Strengthening the Reporting of Observational Studies in Epidemiology (STROBE) reporting guideline.^13^ Information was collected according to the pronouncement of the Central Epidemic Command Center and in accordance with Article 17 of the Communicable Disease Control Act.^14^ As part of the public health response functions of the Central Epidemic Command Center for surveillance purposes, institutional review board approval of this study and informed consent were waived. Prior to analysis, the data were deidentified.

### Ascertainment of Cases

A confirmed case met the criteria of notification for COVID-19 in Taiwan and tested positive by real-time reverse transcriptase–polymerase chain reaction (RT-PCR) test.^15^ Detailed information, including demographic and clinical data, was reported to the National Notifiable Disease Surveillance System.^16^ The investigation team determined the clinical severity of the confirmed patients following the World Health Organization (WHO) interim guidance.^17^

### Contact Tracing for COVID-19

When a patient was laboratory-confirmed to have SARS-CoV-2 infection, a thorough epidemiological investigation, including contact tracing, was implemented by the outbreak investigation team of the Taiwan CDC and local health authorities. The period of investigation started at the date at symptom onset (and could be extended to up to 4 days before symptom onset when epidemiologically indicated) and ended at the date at COVID-19 confirmation. For asymptomatic confirmed cases, the period of investigation was based on the date at confirmation (instead of date at onset) and was determined according to epidemiological investigation. The definition of a close contact was a person who did not wear appropriate personal protection equipment (PPE) while having face-to-face contact with a confirmed case for more than 15 minutes during the investigation period. A contact was listed as a household contact if he or she lived in the same household with the index case. Those listed as family contacts were family members not living in the same household.

For health care settings, medical staff, hospital workers, and other patients in the same setting were included; close contact was defined by contacting an index case within 2 m without appropriate PPE and without a minimal requirement of exposure time. Whether the PPE was regarded as “appropriate” depended on the exposure setting and the procedures performed. For example, for physicians who performed aerosol-generating procedures, such as intubation, an N95 respirator was required. For such procedures, a surgical mask would not be appropriate PPE. Accordingly, the medical staff would be listed as a close contact.

All close contacts were quarantined at home for 14 days after their last exposure to the index case. During the quarantine period, any relevant symptoms (fever, cough, or other respiratory symptoms) of close contacts would trigger RT-PCR testing for COVID-19. For high-risk populations, including household and hospital contacts, RT-PCR was performed regardless of symptoms. Essentially, these high-risk contacts were tested once when they were listed as a close contact. If the initial COVID-19 test result was negative, further testing would only be performed if a close contact developed symptoms during quarantine. The Taiwan CDC used an electronic tracing system (Infectious Disease Contact Tracing Platform and Management System) to follow and record the daily health status of those quarantined contacts.^18^ The information collected included age, sex, the index case, date at exposure, and the exposure setting.

### Data Processing and Analysis

Paired data of index case and close contacts were extracted from the contact tracing database and outbreak investigation reports. For a family cluster, the index case was determined based on the temporality of symptom onset and review of the epidemiological link. A secondary case was excluded from the paired data if the beginning of exposure was after symptom onset of the secondary case (only applied when the secondary case was symptomatic). For health care contacts, the date at exposure would be the date at admission of the case if the exact date at exposure was not recorded.

Incubation period and serial interval were estimated using the contact tracing data in Taiwan and publicly available data sets globally (eMethods in the Supplement). We used the Bayesian hierarchical model to increase the stability in small-sample estimation. The exposure window period was defined as the period between the first and last day of reported exposure to the index case based on contact investigation. Following the WHO, we defined the secondary clinical attack rate as the ratio of symptomatic confirmed cases among the close contacts.^19^ We analyzed the dynamic change of secondary clinical attack rate in relation to symptom onset of the index case (days <0, 0-3, 4-5, 6-7, 8-9, or >9).

The percentage of missing information was small (7.0% for age, 6.1% for sex, and 3.3% for time from onset to exposure; Table 1). In the univariable analysis of secondary clinical attack rate by different exposure characteristics (eg, age), close contacts with missing information in that particular exposure attribute were excluded. All statistical tests were 2-sided with an α level of .05. All confidence intervals (CIs) were 95%. R software (R Foundation for Statistical Computing) and RStan (Stan Development Team) were used for data management and analysis.

**Table 1.** Characteristics of the 2761 Close Contacts by Different Exposure Settings

	Exposure, No. (%)			
	Household (n = 151)	Family (n = 76)	Health care (n = 698)	Others (n = 1836)^a^
Age, median (range), y	33 (1-96)	45 (0**-**88)	39 (0-92)	35 (0-89)
Age group, y				
0-19	24 (16)	14 (18)	29 (4)	214 (12)
20-39	55 (36)	16 (21)	281 (40)	809 (44)
40-59	38 (25)	24 (32)	175 (25)	557 (30)
≥60	26 (17)	11 (14)	119 (17)	175 (10)
Unknown	8 (5)	11 (14)	94 (13)	81 (4)
Sex				
Female	70 (46)	41 (54)	454 (65)	872 (47)
Male	81 (54)	30 (39)	228 (33)	816 (44)
Unknown	0	5 (7)	16 (2)	148 (8)
Time from onset to exposure, median (range), d^b^	−4 (−4 to 9)	6 (−4 to 26)	1 (−4 to 23)	2 (−4 to 26)
Time from onset to exposure, d^b^				
<0	100 (66)	10 (13)	236 (34)	389 (21)
0-3	39 (26)	15 (20)	150 (21)	663 (36)
4-5	6 (4)	6 (8)	38 (5)	166 (9)
6-7	4 (3)	10 (13)	17 (2)	88 (5)
8-9	2 (1)	3 (4)	110 (16)	334 (18)
>9	0	24 (32)	146 (21)	114 (6)
Unknown	0	8 (11)	1 (0.1)	82 (5)

^a^ Others include friends, airline crew members and passengers, and other casual contacts.

^b^ Defined as the elapsed time between the date at symptom onset of the index case and the first date at exposure. For example, people from the group “<0 days” had their first contact with the index case before the case had any symptoms.

## Results

As of March 18, 2020, there were 100 patients with laboratory-confirmed COVID-19 in Taiwan, including 10 clusters of patients and 9 asymptomatic patients. The median age of the 100 patients was 44 years old (range, 11-88 years); 44 were men and 56 were women. Of the 2761 close contacts that were identified, 5.5% were household contacts, 2.8% were non-household family contacts, and 25.3% were health care contacts (Table 1). Through contact tracing, 23 secondary cases were found. One of the 23 cases was excluded from subsequent transmission-pair analysis because the documented day at exposure occurred after symptom onset of the secondary case. None of the 9 asymptomatic case patients transmitted a secondary case. Using the data on the 22 paired cases, we estimated that the median incubation period was 4.1 days (95% credible interval [CrI], 0.4-15.8), and the median serial interval was 4.1 days (95% CrI, 0.1-27.8) (eTables 1-5 and eFigures 1-5 in the Supplement).

**Figure 1.**
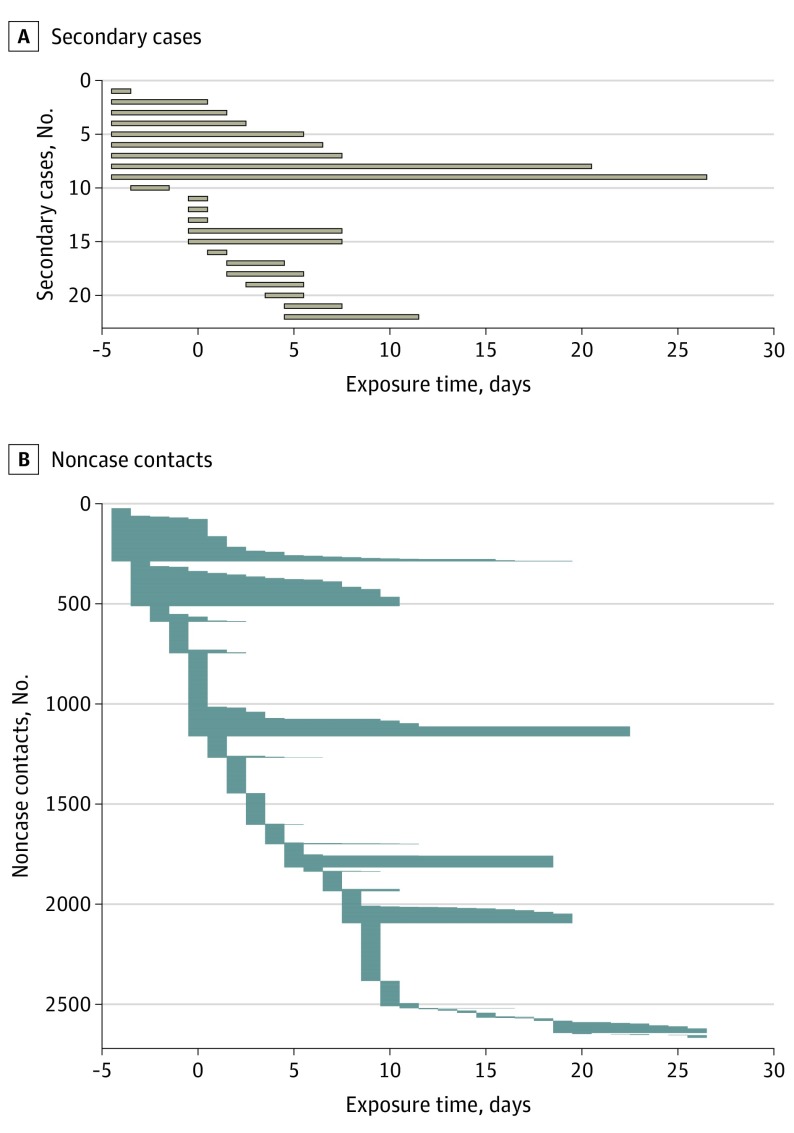
Exposure Window Period Among Secondary Cases and Noncase Contacts The exposure time was defined as the period from the first day of exposure to the index case to the last day of exposure. Time zero indicates the day of symptom onset of the index case.

**Figure 2.**
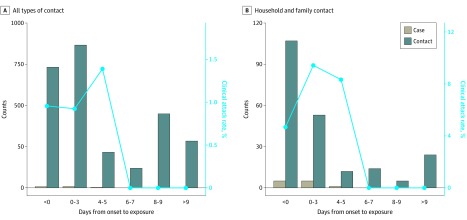
Number of Contacts, Secondary Cases, and Secondary Clinical Attack Rate by the Time of First Exposure

Among the 2761 close contacts, 22 secondary cases of COVID-19 infection (including 4 asymptomatic infections) were detected, with an infection risk of 0.8% (95% CI, 0.5%-1.2%). The secondary clinical attack rate was 18 of 2761, or 0.7% (95% CI, 0.4%-1.0%). Figure 1 shows the exposure window of all contacts. All of the 22 secondary cases had their first exposure before the sixth day of the index case’s symptom onset. By comparison, only 68% of noncase contacts had their first exposure before day 6 (Table 1). The secondary clinical attack rate was higher among those whose initial exposure to the index case was within 5 days of symptom onset than those who were exposed after day 6 (zero transmission of 852 contacts [95% CI, 0%-0.4%]) (Table 2 and Figure 2A). The 735 contacts whose initial exposure occurred before symptom onset of the index case were also at risk, with a secondary clinical attack rate of 1.0% (95% CI, 0.5%-2.0%). A subgroup of 299 contacts with exclusive presymptomatic exposures were also at risk (secondary clinical attack rate, 0.7% [95% CI, 0.2%-2.4%]).

**Table 2.** Secondary Clinical Attack Rate for COVID-19 Among the 2761 Close Contacts by Different Exposure Settings, Times, and Characteristics

	No. of secondary cases (asymptomatic case)	No. of contacts	Secondary clinical attack rate, % (95% CI)	Risk ratio (95% CI)
Exposure setting				
Household	10 (3)	151	4.6 (2.3-9.3)	1 [Reference]
Nonhousehold family	5 (1)	76	5.3 (2.1-12.8)	1.14 (0.34-3.76)
Health care	6 (0)	698	0.9 (0.4-1.9)	0.19 (0.06-0.54)
Others^a^	1 (0)	1836	0.1 (0-0.3)	0.01 (0-0.09)
Time from onset to exposure, d^b^				
<0	10 (3)	735	1.0 (0.5-2.0)	1 [Reference]
0-3	9 (1)	867	0.9 (0.5-1.8)	0.97 (0.35-2.66)
4-5	3 (0)	216	1.4 (0.5-4.0)	1.46 (0.38-5.59)
6-7	0	119	0 (0-3.1)	0
8-9	0	449	0 (0-0.9)	0
>9	0	284	0 (0-1.3)	0
Exclusively presymptomatic exposure^c^				
No	20 (4)	2371	0.7 (0.4-1.1)	1 [Reference]
Yes	2 (0)	299	0.7 (0.2-2.4)	0.99 (0.23-4.29)
Age of close contacts, y				
0-19	1 (1)	281	0 (0-1.4)	0
20-39	8 (2)	1161	0.5 (0.2- 1.1)	1 [Reference]
40-59	10 (1)	794	1.1 (0.6-2.1)	2.19 (0.78-6.14)
≥60	3 (0)	331	0.9 (0.3-2.6)	1.75 (0.44-6.97)
Source of index case				
Local	18 (3)	967	1.6 (1.0-2.5)	1 [Reference]
Imported	4 (1)	1794	0.2 (0.1-0.5)	0.11 (0.03-0.37)
Clinical severity of index case				
Asymptomatic	0	91	0 (0-4.1)	0
Mild illness	4 (0)	1097	0.4 (0.1-0.9)	1 [Reference]
Pneumonia				
Mild	5 (2)	761	0.4 (0.1-1.2)	1.08 (0.24-4.82)
Severe	7 (0)	511	1.4 (0.7-2.8)	3.76 (1.10-12.76)
ARDS/sepsis	6 (2)	275	1.5 (0.6-3.7)	3.99 (1.00-15.84)

Abbreviations: ARDS, acute respiratory distress syndrome; COVID-19, coronavirus disease 2019.

^a^ Others include friends, airline crew members and passengers, and other casual contacts.

^b^ Defined as the elapsed time between the date at symptom onset of the index case and the first date at exposure. For example, people from the group “<0 days” had their first contact with the index case before the case had any symptoms.

^c^ All the reported exposures occurred during the presymptomatic period of the index case.

The secondary clinical attack rate was 4.6% (95% CI, 2.3%-9.3%) among 151 household contacts and 5.3% (95% CI, 2.1%-12.8%) in 76 nonhousehold family contacts (Table 2). The high attack rate from early exposure remained when the analysis was restricted to household and nonhousehold family contacts (Table 3 and Figure 2B). The attack rates were higher among those aged 40 to 59 years (1.1% [95% CI, 0.6%-2.1%]) and those aged 60 years and older (0.9% [95% CI, 0.3%-2.6%]). The 786 close contacts of the 6 confirmed cases presenting with severe disease were at a higher risk compared with the 1097 close contacts of the 56 cases presenting with mild disease (risk ratio, 3.76 [95% CI, 1.10-12.76] and 3.99 [95% CI, 1.00-15.84] for severe pneumonia and acute respiratory distress syndrome/sepsis, respectively). Among the 91 close contacts of the 9 asymptomatic cases, no secondary transmission was observed. The secondary attack rate among contacts of cases with infection acquired in Taiwan was higher than that among contacts of cases with infection acquired outside of Taiwan (Table 2).

**Table 3.** Risk for Symptomatic COVID-19 Infection Among the 2761 Close Contacts, Simultaneously Stratified by Exposure Setting and Time From Symptom Onset of the Index Case to First Day of Exposure

First day of exposure, d	Household		Nonhousehold family		Health care		Others^a^	
	Case/contact, No.	Attack rate, % (95% CI)^b^	Case/contact, No.	Attack rate, % (95% CI)^b^	Case/contact, No.	Attack rate, % (95% CI)^b^	Case/contact, No.	Attack rate, % (95% CI)^b^
<0	4/100	4.0 (1.6-9.8)	1/10	10.0 (1.8-40.4)	2/236	0.8 (0.2-3.0)	0/389	0 (0-1.0)
0-3	2/39	5.1 (1.4-16.9)	3/15	20.0 (7.0-45.2)	3/150	2.0 (0.7-5.7)	0/663	0 (0-0.6)
4-5	1/6	16.7 (3.0-56.4)	0/6	0 (0-39.0)	1/38	2.6 (0.5-13.5)	1/166	0.6 (0.1-3.3)
6-7	0/4	0 (0-49.0)	0/10	0 (0-27.8)	0/17	0 (0-18.4)	0/88	0 (0-4.2)
8-9	0/2	0 (0-65.7)	0/3	0 (0-56.1)	0/110	0 (0-3.3)	0/334	0 (0-1.1)
>9	0/0	NC	0/24	0 (0-13.8)	0/146	0 (0-2.6)	0/114	0 (0-3.3)

Abbreviations: COVID-19, coronavirus disease 2019; NC, not calculable.

^a^ Others include friends, airline crew members and passengers, and other casual contacts.

^b^ Secondary clinical attack rate.

## Discussion

Our analysis of close contacts to confirmed COVID-19 cases revealed a relatively short infectious period of COVID-19 and a higher transmission risk around the time of symptom onset of the index case, followed by a lower transmission risk at the later stage of disease. The observed decreasing transmission risk over time for COVID-19 was in striking contrast to the transmission pattern of severe acute respiratory syndrome (SARS), in which the transmission risk remained low until after day 5 of symptom onset in the index cases.^20^ Our study and the study by Nishiura et al^4^ revealed a short serial interval of COVID-19, with a median of 4 to 5 days. In contrast, the mean serial interval of SARS was estimated to be 8.4 days in Singapore.^20^ The present contact tracing analysis suggested that the shorter serial interval of COVID-19 was due to the combination of early-stage transmission and a short period of infectiousness.

The observed pattern of the secondary clinical attack rate over time was also consistent with the quantitative data of the SARS-CoV-2 viral shedding in upper respiratory specimens, which has been found in China to be a high viral load around the time of symptom onset, followed by a gradual decrease in viral shedding to a low level after 10 days.^5^ The viral load was similar among asymptomatic, minimally symptomatic, and symptomatic patients. Another virological study in patients with COVID-19 in Germany also found no viable isolates of the virus after the first week of symptoms.^21^ Our findings agree with the virological data on high transmissibility of COVID-19 in the first week after the onset of symptoms and decreased risk afterwards.^21^ We also documented and quantified the transmission potential of COVID-19 in a subgroup of contacts whose exposure occurred exclusively during the presymptomatic period of the index case. Our analysis revealed a similar clinical attack rate between the contacts who only had presymptomatic exposure and those who had postsymptomatic exposure.

To summarize the evidence, the decreasing risk for secondary infection over time in our study, the observed short serial interval, and the trend of decreasing viral shedding and viability after symptom onset strongly suggested high transmissibility of the disease near or even before the day of symptom onset. Because the onset of overt clinical symptoms, such as fever, dyspnea, and signs of pneumonia, usually occurred 5 to 7 days after initial symptom onset, the infection might well have been transmitted at or before the time of detection.^22,23^ This characteristic makes containment efforts challenging. In a modeling study, Hellewell et al^24^ found that the possibility of controlling COVID-19 through isolation and contact tracing decreased with increasing proportion of transmission that occurred before symptom onset. The findings of this modeling study, when viewed in the context of our findings, might help to explain the difficult situation in such areas and countries as China, South Korea, Iran, and Italy. Aggressive social distancing and proactive contact tracing might be necessary to block the transmission chain of COVID-19 and to keep presumptive patients away from susceptible populations with a high risk for severe disease.

The observed short duration of infectiousness with lower risk of transmission 1 week after symptom onset has important implications for redirecting the efforts to control COVID-19. Given the nonspecific and mostly mild symptoms of COVID-19 at presentation, patients are often identified and hospitalized at a later stage of disease when the transmissibility of infection has started to decrease. In this case, hospitalization would not be helpful for isolation and reducing transmission, and should be only for patients whose clinical course is sufficiently severe. When the number of confirmed cases rapidly increases, home care for patients with mild illness may be preferred.^25^ In Taiwan (where patients with COVID-19 have been routinely hospitalized), the most prolonged duration of hospital isolation for the 100 confirmed cases was more than 2 months. If every patient with mild illness is to be isolated in the hospital or other isolation facilities for such a prolonged period during a large epidemic, the health care system would soon be overwhelmed, and the case-fatality rate may increase, as observed in Wuhan.^26,27^ Similarly, better understanding of the potential duration of transmission could help direct containment strategies. For example, contact tracing could focus on the contacts near or even before symptom onset of the index cases when the number of index cases or contacts is too large for all contacts to be traced, given the available resources.

Several patients in our study were initially considered to have pneumonia of unknown etiology and had multiple contacts in the health care setting before being diagnosed. However, the number of health care contacts that led to nosocomial transmission was low. Besides the basic PPE used by medical staffs, this finding might be due to the late admissions of these patients and their lower risk of transmitting COVID-19 by the time of hospitalization. This pattern is compatible with the observations in China and Hong Kong. In China, the number of nosocomial infections might be lower than reported because some health care workers acquired infections in their households rather than in the health care faciltiy.^9^ In Hong Kong, most hospitalization was also delayed to at least 5 days after disease onset.^28^ In closed settings such as a hospital or a cruise ship,^29,30^ fomite transmission might play an important role, amplifying the risk of transmission and making the temporality of transmission less identifiable.^30,31,32^ Better understanding of the dynamic change of transmissibility of COVID-19 over time and how health care workers are most likely to be infected could allow for better targeting of control measures, including the use of appropriate PPE.

In the contact tracing cohort, we observed a relatively low transmission rate of COVID-19. During the study period (January to early March 2020), the major containment measures in Taiwan were travel alerts with restriction to affected countries (principally China), home quarantine for travelers entering Taiwan, and comprehensive contact tracing for confirmed cases.^11^ In response to a possible shortage of face masks, the government proactively initiated a name-based rationing system for mask purchase and boosted the production of face masks to ensure the availability for both N95 respirators and face masks to both health care professionals and the general public. A general recommendation on social distancing from the government was not in place, but spontaneous behavioral changes that reduced community mobility were observed.^33^

### Limitations

Our study has limitations. First, we did not completely examine contacts before the symptom onset of the index cases. Therefore, we might have underestimated the importance of early transmission. Thus, the actual contribution of early transmission to new infections could be greater than our estimates suggests. Our findings agree with the recommendation from the WHO to use 4 days before symptom onset as the starting date for contact tracing.^19^ This modification may help to further understand the pattern of early transmission in COVID-19. Second, we could not completely separate out the effect of close household contact and early contact given the strong correlation of the 2. The increased transmissibility in the early stage of COVID-19 may be partially attributed to the effect of household and nonhousehold family contacts rather than increased infectiousness at the early stage. When we stratified by type of exposure, however, the pattern of early transmission remained.

## Conclusions

In summary, the findings of this study suggest that most transmission of COVID-19 occurred at the very early stage of the disease or even before the onset of symptoms, and the secondary clinical attack rate among contacts decreased over time as symptoms developed and progressed. The pattern of high transmissibility near and before symptom onset and the likely short infectious period of the virus could inform control strategies for COVID-19, as well as additional studies to fully elucidate the transmission dynamics of the virus.

## Notes

Supplement. eMethods. Serial interval and incubation period distribution **eTable 1.** The estimated distribution parameters of the serial interval for each exposure setting (household, community, and others) **eTable 2.** The estimated distribution parameters of the serial interval for each region (Taiwan, Mainland China, and others) **eTable 3.** The estimated distribution parameters of the serial interval (overall countries and settings) **eTable 4.** The estimated distribution parameters of the incubation period for each region (Taiwan, Mainland China, and others) **eTable 5. **The estimated distribution parameters of the incubation period (overall countries and settings) **eFigure 1.** The estimated serial interval distributions for each exposure setting (household, community, and others) **eFigure 2.** The estimated serial interval distributions for each region (Taiwan, Mainland China, and others) **eFigure 3.** The estimated serial interval distribution (overall countries and settings) **eFigure 4.** The estimated incubation period distributions for each region (Taiwan, Mainland China, and others) **eFigure 5.** The estimated incubation period distribution (overall countries and settings) **eReferences**
