# Supplementary material for: Contact Tracing Assessment of COVID-19 Transmission Dynamics in Taiwan and Risk at Different Exposure Periods Before and After Symptom Onset
Source: JAMA Intern Med. 2020 May 1;180(9):1156–63. doi: 10.1001/jamainternmed.2020.2020 (PMC7195694; doi:10.1001/jamainternmed.2020.2020)
Supplement: Supplement. — eMethods. Serial interval and incubation period distribution eTable 1. The estimated distribution parameters of the serial interval for each exposure setting (household, community, and others) eTable 2. The estimated distribution parameters of the serial interval for each region (Taiwan, Mainland China, and others) eTable 3. The estimated distribution parameters of the serial interval (overall countries and settings) eTable 4. The estimated distribution parameters of the incubation period for each region (Taiwan, Mainland China, and others) eTable 5. The estimated distribution parameters of the incubation period (overall countries and settings) eFigure 1. The estimated serial interval distributions for each exposure setting (household, community, and others) eFigure 2. The estimated serial interval distributions for each region (Taiwan, Mainland China, and others) eFigure 3. The estimated serial interval distribution (overall countries and settings) eFigure 4. The estimated incubation period distributions for each region (Taiwan, Mainland China, and others) eFigure 5. The estimated incubation period distribution (overall countries and settings) eReferences [file jamainternmed-e202020-s001.pdf]

Supplementary Online Content

Cheng H-Y, Jian S-W, Liu D-P, Ng T-C, Huang W-T, Lin H-H; Taiwan COVID-19 Outbreak Investigation Team. Contact tracing assessment of COVID-19 transmission dynamics in Taiwan and risk at different exposure periods before and after symptom onset. Published online May 1, 2020. *JAMA Internal Medicine*. doi:10.1001/jamainternmed.2020.2020

eMethods. Serial interval and incubation period distribution

eTable 1. The estimated distribution parameters of the serial interval for each exposure setting (household, community, and others)

eTable 2. The estimated distribution parameters of the serial interval for each region (Taiwan, Mainland China, and others)

eTable 3. The estimated distribution parameters of the serial interval (overall countries and settings)

eTable 4. The estimated distribution parameters of the incubation period for each region (Taiwan, Mainland China, and others)

eTable 5. The estimated distribution parameters of the incubation period (overall countries and settings)

eFigure 1. The estimated serial interval distributions for each exposure setting (household, community, and others)

eFigure 2. The estimated serial interval distributions for each region (Taiwan, Mainland China, and others)

eFigure 3. The estimated serial interval distribution (overall countries and settings)

eFigure 4. The estimated incubation period distributions for each region (Taiwan, Mainland China, and others)

eFigure 5. The estimated incubation period distribution (overall countries and settings)

eReferences

This supplementary material has been provided by the authors to give readers additional information about their work.

© 2020 Cheng H-Y et al. *JAMA Internal Medicine.*

eMethods. Serial interval and incubatiuon period distribution

Serial interval distribution

Data

We collated the symptom onset dates of COVID-19 patients with confirmed transmission links in published papers, news reports and press releases from local governments. In total, there are 58 transmission pairs where both infectors and infectees developed symptoms after the infection. The exposure settings were categorized into (1) household, (2) community and, (3) others (including workplace, friend, family, and unclear setting). Countries were categorized in to (1) Taiwan, (2) Mainland China, and (3) Others.

Methods

We adopted the statistical method by Backer, et al[2] to deal with uncertain interval data. A gamma distribution was fitted to the observed serial intervals (SI) in a Bayesian framework. For each stratum $k = 1, 2, 3$,

$$SI^k \sim Gamma(\alpha^k, \beta^k)$$
$$\alpha^k \sim Exponential(1/\theta^{shape})$$
$$\beta^k \sim Exponential(1/\theta^{scale})$$

where each stratum (k) has its own serial interval distribution. The stratum-specific distribution parameters, shape (α^k) and scale (β^k) are related by an exponential hyper-parameter distribution, with mean θ^{shape}, and θ^{scale} respectively. Non-informative priors for the hyper-parameters were used.

$$\theta^{shape} \sim Half - normal\ (0, 1000)$$
$$\theta^{scale} \sim Half - normal\ (0, 1000)$$

Since the reported symptom onset dates can be intervals, we set uniform priors for the onset intervals, and estimated the symptom onset dates. For each individual $i = 1, \dots, n$

$$X_i^0 \sim Uniform(XL_i^0, XR_i^0)$$
$$X_i^1 \sim Uniform(XL_i^1, XR_i^1)$$
$$SI_i = X_i^1 - X_i^0$$

where $[XL_i^0, XR_i^0]$ is the symptom onset interval for the infector in pair i, and $[XL_i^1, XR_i^1]$ is the symptom onset interval for the infectee. We ran 4 MCMC chains using the NUTS algorithm in *Stan*. Each chain contains 1000 warmup iterations and 500 samples, resulting in a total 2000 samples.

Incubation period distribution

Data and methods

Apart from the 18 symptomatic cases with known exposure periods in Taiwan, we included publicly available, crowd-sourced dataset provided by Sun,

© 2020 Cheng H-Y et al. *JAMA Internal Medicine.*

et al[3]. Only cases with known exposure windows (both beginning and end) and the dates of symptom onset were included, because the results were sensitive to ad hoc beginning dates of exposure. In total, 65 cases were included in the analysis of the incubation period. We applied same statistical methods as in the estimation of the serial interval distribution, by substituting XL_i^0 and XR_i^0 by the lower and upper bounds of the exposure period EL_i^1 and ER_i^1 in the previous model.

© 2020 Cheng H-Y et al. *JAMA Internal Medicine.*

eTable 1. The estimated distribution parameters of the serial interval for each exposure setting (household, community, and others), with 95% credible interval (CrI).

**Setting**	**Median estimate (95% CrI)**	**Shape (95% CrI)**	**Scale (95% CrI)**
Household	5.23 (0.74, 17.56)	1.96 (1.15, 3.15)	3.19 (1.96, 5.94)
Community	2.89 (0, 37.34)	0.45 (0.17, 0.99)	15.81 (5.25, 90.68)
Others	3.26 (0.1, 18.22)	0.94 (0.56, 1.49)	5.11 (2.71, 10.55)

eTable 2. The estimated distribution parameters of the serial interval for each region (Taiwan, Mainland China, and others), with 95% credible interval (CrI).

**Setting**	**Median estimate (95% CrI)**	**Shape (95% CrI)**	**Scale (95% CrI)**
Taiwan	4.1 (0.06, 27.78)	0.77 (0.43, 1.27)	8.75 (4.49, 21.38)
China	5.03 (1.46, 12.13)	3.9 (1.63, 8.2)	1.41 (0.63, 3.74)
Others	2.89 (0.04, 20.64)	0.72 (0.44, 1.14)	6.71 (3.66, 14.09)

eTable 3. The estimated distribution parameters of the serial interval (overall countries and settings), with 95% credible interval (CrI).

**Setting**	**Median estimate (95% CrI)**	**Shape (95% CrI)**	**Scale (95% CrI)**
Overall	4.11 (0.19, 20.39)	1.09 (0.77, 1.46)	5.26 (3.61, 8.4)

© 2020 Cheng H-Y et al. *JAMA Internal Medicine.*

eTable 4. The estimated distribution parameters of the incubation period for each region (Taiwan, Mainland China, and others), with 95% credible interval (CrI).

**Setting**	**Median estimate (95% CrI)**	**Shape (95% CrI)**	**Scale (95% CrI)**
Taiwan	4.08 (0.39, 15.8)	1.55 (0.73, 2.93)	3.32 (1.6, 8.79)
China	6.01 (2.11, 13.11)	5.07 (2.99, 8.75)	1.27 (0.68, 2.29)
Others	6.1 (1.51, 15.95)	3.23 (2.34, 4.59)	2.1 (1.44, 3.08)

eTable 5. The estimated distribution parameters of the incubation period (overall countries and settings), with 95% credible interval (CrI).

**Setting**	**Median estimate (95% CrI)**	**Shape (95% CrI)**	**Scale (95% CrI)**
Overall	5.82 (1.39, 15.51)	3.09 (2.37, 3.96)	2.1 (1.59, 2.86)

© 2020 Cheng H-Y et al. *JAMA Internal Medicine.*

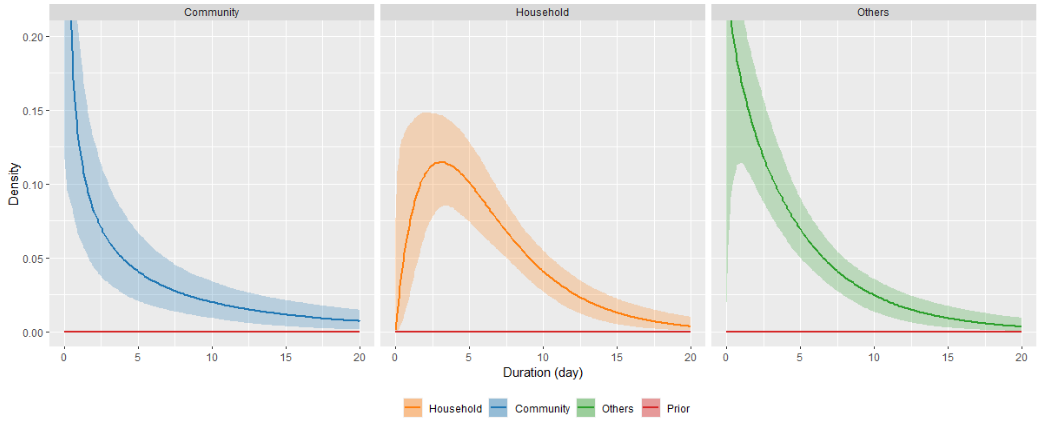

eFigure 1. The estimated serial interval distributions for each exposure setting (household, community, and others), in solid lines. The shaded areas represent 95% credible interval (CrI).

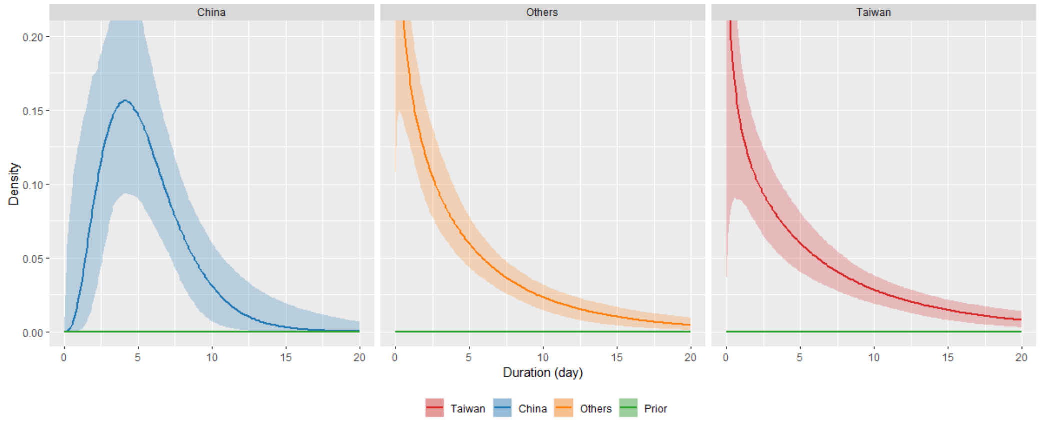

eFigure 2. The estimated serial interval distributions for each region (Taiwan, Mainland China, and others), in solid lines. The shaded areas represent 95% credible interval (CrI).

© 2020 Cheng H-Y et al. *JAMA Internal Medicine.*

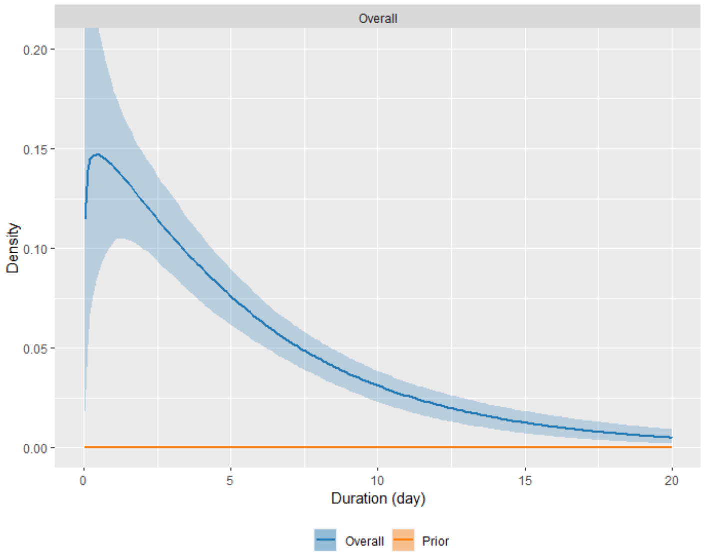

eFigure 3. The estimated serial interval distribution (overall countries and settings), in solid lines. The shaded areas represent 95% credible interval (CrI).

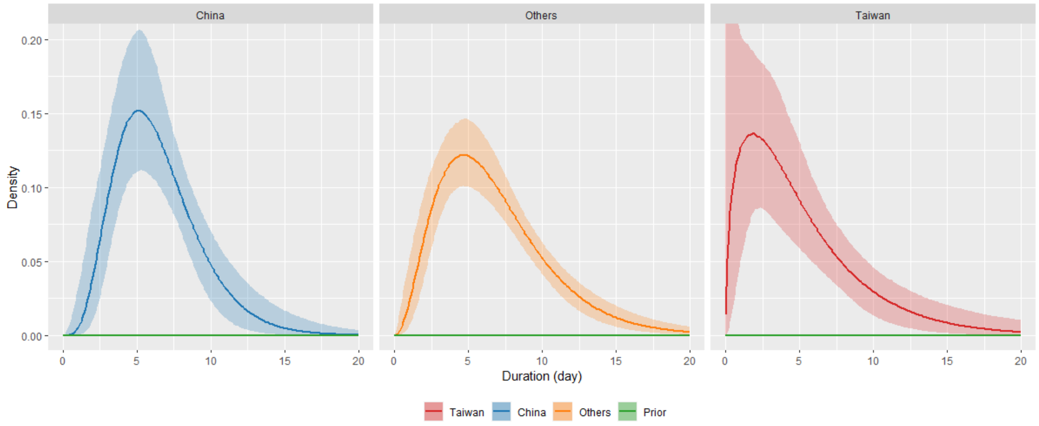

eFigure 4. The estimated incubation period distributions for each region (Taiwan, Mainland China, and others), in solid lines. The shaded areas represent 95% credible interval (CrI).

© 2020 Cheng H-Y et al. *JAMA Internal Medicine.*

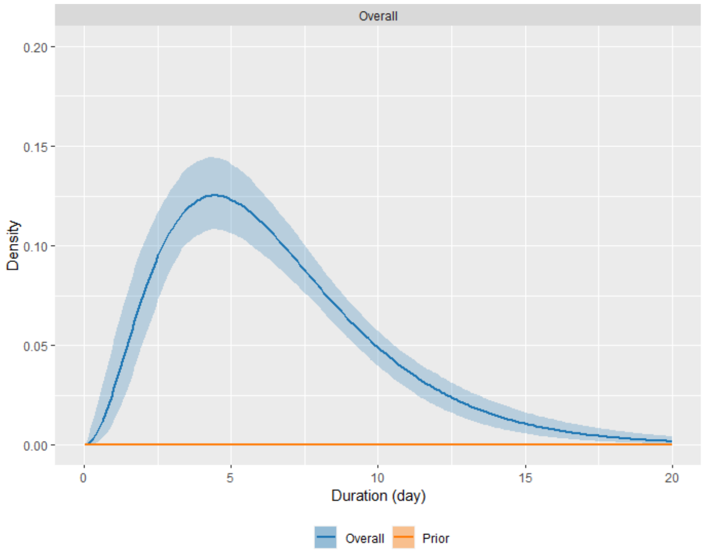

eFigure 5. The estimated incubation period distribution (overall countries and settings), in solid lines. The shaded areas represent 95% credible interval (CrI).

© 2020 Cheng H-Y et al. *JAMA Internal Medicine.*

eReferences

1 Carpenter, B. *et al.* Stan: A Probabilistic Programming Language. *2017* **76**, Journal of Statistical Software, doi:10.18637/jss.v076.i01 (2017).

2 Backer, J. A., Klinkenberg, D. & Wallinga, J. Incubation period of 2019 novel coronavirus (2019-nCoV) infections among travellers from Wuhan, China, 20–28 January 2020. *Eurosurveillance* **25** (2020).

3 Sun, K., Chen, J. & Viboud, C. Early epidemiological analysis of the coronavirus disease 2019 outbreak based on crowdsourced data: a population-level observational study. *The Lancet Digital Health*, doi:https://doi.org/10.1016/S2589-7500(20)30026-1 (2020).

© 2020 Cheng H-Y et al. *JAMA Internal Medicine.*
